# Continuing Professional Development ‐ Medical Imaging

**DOI:** 10.1002/jmrs.829

**Published:** 2024-10-09

Maximise your continuing professional development (CPD) by reading the following selected article and answer the five questions. Please remember to self‐claim your CPD and retain your supporting evidence. Answers will be available via the QR code and published in JMRS – Volume 72, Issue 4, December 2025.

## Medical Imaging – Original Article

### Demonstrating the use of population level data to investigate trends in the rate, radiation dose and cost of Computed Tomography across clinical groups: Are there any areas of concern?

Kamarova S , Youens D , Ha NT , Bulsara M , Doust J , Fox R , Kritz M , McRobbie D , O'Leary P , Parizel PM , Slavotinek J , Wright C , Moorin R . (2025) J Med Radiat Sci. 10.1002/jmrs.811 PMC1244960538982690

1. In this study, index events (i.e. events that indicated the diagnosis of a new condition) were classified based on which of the following data collections?
  - Hospital admissions and emergency department presentations
  - Hospital admissions, emergency department presentations and Medicare claims
  - Death registrations, hospital admissions and emergency department presentations
  - Medicare claims and Picture Archiving Communications System records
2. Why did the authors of this study exclude individuals who died within 90 days following the index event?
  - Medicare records were not available for these individuals.
  - Radiation exposure from CT is not a concern for those near the end of life.
  - There was limited follow‐up to assess CT use for these individuals.
  - The cause of death may not have been related to the broad diagnostic chapter determined from the index event.
3. In this study, what major clinical chapter was ranked highest for both cumulative effective dose and associated cancer risks across the study years, with no significant increase in costs?
  - Digestive system
  - Neoplasms
  - Blood and blood forming organs
  - Injury and poisoning
4. Which major clinical chapter recorded the largest percentage increase in mean cumulative effective dose from 2006 to 2015?
  - Endocrine, nutritional and metabolic disorders
  - Skin and connective tissues
  - Digestive system
  - Respiratory system
5. The authors of this study propose that one possible reason for the increase in CT scans during the study period is the restrictions on the use of which other imaging modality in Australia?
  - X‐ray
  - Positron emission tomography
  - Magnetic resonance imaging
  - Ultrasound

## Answers

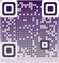

Scan this QR code to find the answers.
